# Successful Aging

**DOI:** 10.1155/2012/438537

**Published:** 2012-12-09

**Authors:** Loretta DiPietro, Maria Fiatarone Singh, Roger Fielding, Hiroshi Nose

**Affiliations:** ^1^School of Public Health and Health Services, The George Washington University, Washington, DC 20052, USA; ^2^Faculty of Health Sciences, University of Sydney, Sydney, NSW 2000, Australia; ^3^Nutrition, Exercise Physiology Laboratory, Jean Mayer USDA Human Nutrition Research Center on Aging at Tufts University, Boston, MA 02111, USA; ^4^Department of Sports Medical Sciences, Institute of Pathogenesis and Disease Prevention, Shinshu University Graduate School of Medicine, 3-1-1 Asahi, Matsumoto, Nagano 390-8621, Japan

Advances in public health and in health care are keeping people alive longer, and consequently, the proportion of older people in the global population is increasing rapidly. In the United States, persons aged 65 years and older comprise about 13% of the population, and their numbers are projected to reach 72.1 million (19% of the total) by the year 2030—a twofold increase over the older adult population in 2000 [1]. Perhaps of greatest interest in aging research is the rise in the “oldest old” segment of the population—those persons aged 85 years and older. Since 1930, this demographic subgroup has doubled in number every 30 years and is projected to be the fastest growing sector of the older population well into the 21st century [1]. This shifting demographic trend has substantial political, social, medical, and economic implications for most of the world.

Physiological function declines with aging, even among the most robust sectors of the older population. The degree to which this decline is attributable to true biological aging and the degree to which it is attributable to social or lifestyle factors that accompany older age is not entirely clear. Evidence suggests, however, that there is substantial heterogeneity in patterns of aging [2]. That is, while many older people continue to show expected patterns of decline in health and functional ability, others appear more resilient to various physiological (e.g., infection), emotional (e.g., bereavement), or environmental challenges. Thus, *resiliency* to various challenges or perturbations can be considered an underlying hallmark of “successful aging,” which is the focus of this special issue.

Rowe and Kahn [3] first developed a model to characterize those very robust and independent older persons according to three domains: (1) disease risk; (2) physical or cognitive capacity; (3) engagement with life. Most newer models of successful aging now expand these domains to include additional measures of physical (e.g., self-rated health; days in bed; extremity strength; timed 15 ft walk; report of ADL or IADL limitations), cognitive (e.g., Minnesota Mini-Mental Status score), and psychosocial (e.g., Life Satisfaction score, CES-D score; Life View score; perceived economic status) function. Three of the papers in this special issue describe the prevalence of successful aging among various old and very old study populations according to one or more of these models. First, J. Cho et al. compared Rowe and Kahn's original model of successful aging with an alternative psychosocial model comprising aspects of subjective health, perceived economic status, and happiness. The authors observed a significantly greater proportion of octogenarians and centenarians to be characterized as “successful” according to the alternative model and argue that as people succeed into advanced older age (i.e., >80 years), additional criteria are necessary in order to capture the multidimensional aspects defining successful aging. Investigators on the Finnish vitality 90+ study (L. Nosraty et al.) observed that the prevalence of successful aging was greater in men than in women and was associated with being married and with higher level of educational attainment. Indeed, these findings also suggest that models emphasizing simply the absence of disease or disability may not be sensitive enough to capture more important attributes of very old age, such as autonomy, adaptation, or sense of purpose. Finally, using data from older participants in the Cardiovascular Health Study, S. Thielke and P. Diehr examined sex and age differences in the probability of remaining on a successful aging trajectory according to 12 different domains of successful aging. Not surprisingly, the probability of remaining “successful” in most of the domains studied declined significantly as participants aged, and similar to the findings among older Finns, men were more (not less) likely to remain “successful” on the majority of domains compared with women of the same age. Moreover, these same men were more likely than women to transition from “successful” to death, without transitioning to a state of sickness first. This latter finding reflects the “rectangularization” of the survival curve (i.e., compression of morbidity [4]) in successful aging and suggests that its prevalence is greater in men in advanced older age. A fourth study in this volume by G. K. Randall and colleagues is methodological in nature and proposes a shortened and valid version of the Duke Older Americans Resources and Services procedures (OARS) functional assessment tool, thereby reducing the respondent burden among the oldest old. In sum, these descriptive studies continue to challenge and expand our preconceptions of successful aging and, at the same time, provide even more evidence of the elusive and heterogeneous nature of growing really old.

The challenges of describing successful aging, notwithstanding those who study the oldest old, have to contend with the enormous methodological issue of selective survival. That is, those people most susceptible to putative risk factors for chronic disease and disability have not survived into their 8th decade, leaving only the most robust older people available to be studied. This issue becomes even more pronounced when performing research on those living past the age of 100 years—especially if they are men. Consequently, investigators often observe smaller effect sizes than what might be observed in younger people. The only experimental study in this issue, by L. DiPietro et al., examined the relation between stress reactivity and 24 h glycemic control in sedentary, but healthy older people. Peak cortisol responses to the stress challenge were significantly different compared with the control condition; however, the magnitude of response appeared blunted compared with what might be observed in middle-aged populations studied under the same conditions. Also, stress-related disruptions in glycemic control were minimal in this healthy older study sample. Continuous glucose monitoring over 24 h provided evidence that any subtle metabolic disruption (apparent only up to 6 h following the stress challenge) had completely dissipated by 24 h. Interestingly, the issue's only epidemiologic study, which analyzed data from the Canadian Community Health Survey-Healthy Aging supplement (S. Dogra and L. Stathokostas), is among the first to report a significantly elevated and potentially graded odds of successful aging among the least sedentary respondents compared with the most sedentary, independent of level of physical activity. These elevated odds were similar for men and women, and (contrary to several epidemiologic studies of aging in which estimates of relative risk attenuate as people age) the odds of successful aging due to lower amounts of sitting and higher amounts of physical activity were similar between middle-aged (45–65 years) and older (65+ years) respondents.

Finally, results from a systematic literature review on the use of robotics in geriatric care (A. J. Pearce et al.) provide ample evidence of the availability of robotic devices in allowing healthy older people and those with disabilities to remain independent, safe, and socially connected in their community setting. These findings have enormous public health implications as the Aging-in-Place movement gains momentum and as naturally occurring retirement communities (NORCs) continue to grow in the United States and globally. Again, as smart technology evolves and becomes accessible to growing numbers of very old people, our models to describe successful aging will need to evolve as well.

In sum, the papers included in this special volume on successful aging represent an exciting, insightful, and challenging view of this important interdisciplinary field. We hope that this special issue will attract readers with the same scientific and practice interests.
